# Subpopulations of cells from bronchoalveolar lavage can predict prognosis in sarcoidosis

**DOI:** 10.1183/13993003.01450-2019

**Published:** 2020-01-30

**Authors:** Pernilla Darlington, Susanna Kullberg, Anders Eklund, Johan Grunewald

**Affiliations:** 1 Respiratory Medicine Division, Dept of Clinical Science and Education, Södersjukhuset and Karolinska Institutet, Stockholm, Sweden; 2 Respiratory Medicine Division, Dept of Medicine Solna and Center for Molecular Medicine (CMM), Karolinska University Hospital and Karolinska Institutet, Stockholm, Sweden

## Abstract

Sarcoidosis is characterised by an accumulation of CD4^+^ T-cells in the lungs and an increased bronchoalveolar lavage fluid (BALF) CD4/CD8 ratio (>3.5) [1]. In sarcoidosis, an expansion of BALF CD4^+^ T-cells expressing the T-cell receptor Vα2.3 has been associated with good prognosis and with specific HLA-alleles, *i.e.* HLA-DRB1*0301 and HLA-DRB3*0101 (which is often carried together with HLA-DRB1*13). HLA-DRB1*03 and HLA-DRB3*0101 molecules show similarities in the region important for antigen presentation and both may therefore be capable of presenting identical antigens to the lung T-cells [2].

## Abstract 2

**The higher the proportion of a T-cell subset (CD4^+^Vα2.3^+^ T-cells) in bronchoalveolar lavage fluid of sarcoidosis patients, the better the prognosis and therefore it may be used as an additional prognostic tool** http://bit.ly/2Mta0Cs

To the Editor:

Sarcoidosis is characterised by an accumulation of CD4^+^ T-cells in the lungs and an increased bronchoalveolar lavage fluid (BALF) CD4/CD8 ratio (>3.5) [1]. In sarcoidosis, an expansion of BALF CD4^+^ T-cells expressing the T-cell receptor Vα2.3 has been associated with good prognosis and with specific HLA-alleles, *i.e.* HLA-DRB1*0301 and HLA-DRB3*0101 (which is often carried together with HLA-DRB1*13). HLA-DRB1*03 and HLA-DRB3*0101 molecules show similarities in the region important for antigen presentation and both may therefore be capable of presenting identical antigens to the lung T-cells [2]. Furthermore, an expansion defined as >10.5% CD4^+^ Vα2.3^+^ BALF T-cells is commonly seen in patients with Löfgren's syndrome [3], which is characterised by an acute onset with bilateral ankle arthritis and/or erythema nodosum, bilateral hilar lymphadenopathy and, in some cases, with parenchymal infiltrates and usually fever [4]. We have previously shown that very high expansions of CD4^+^ Vα2.3^+^ T-cells are associated with Löfgren's syndrome and a disease duration <2 years [3]. However, not all patients with an expansion of CD4^+^ Vα2.3^+^ T-cells have Löfgren's syndrome and resolving disease. In this much enlarged study on a HLA-typed sarcoidosis cohort, we aimed at investigating the clinical characteristics of patients with an expansion of CD4^+^ Vα2.3^+^ T-cells in BALF and to analyse if the degree of expansion may predict the prognosis of sarcoidosis.

In a registry of sarcoidosis patients (n=661, including 252 with Löfgren's syndrome), all investigated with bronchoscopy and BAL for diagnostic purposes and HLA-typed and followed for at least 2 years, 248 subjects were identified with BALF CD4^+^ Vα2.3^+^ T-cells expansions. An expansion was defined as three times the median percentage of Vα2.3^+^ CD4^+^ T cells in peripheral blood of healthy subjects, as previously described (3×3.5%) [3]. Disease activity was evaluated 2 years after disease onset, considering presence of symptoms (*e.g.* cough, fatigue, dyspnoea, fever), serum-ACE activity, spirometry values and chest radiographic findings. Patients without any pathological findings were regarded to have a resolving disease.

We focused on patients with Vα2.3^+^ CD4^+^ T-cell expansions, out of which 73% had classical Löfgren's syndrome (table 1). They were all judged to have active disease at the time for bronchoscopy. The percentage of Vα2.3^+^ CD4^+^ T-cells in BALF is known to be normalised when the patients recover [5]. All patients were without immunosuppressive treatment at the time for bronchoscopy. After 2 years follow-up very few patients with Löfgren's syndrome, but some more with non-Löfgren's syndrome, had been treated with immunosuppressants. The sarcoidosis diagnosis was made through typical clinical and radiographic manifestations, findings at bronchoscopy with BAL including an elevated CD4/CD8-ratio (>3.5) and/or positive biopsies, in accordance with the criteria of the World Association of Sarcoidosis and other Granulomatous Disorders [6]. Chest radiographs were evaluated as previously described [7]. Written informed consent was obtained from all subjects, and approval was granted from the regional ethical review board.

**TABLE 1:** Clinical characteristics of patients with Vα2.3^+^ T-cells >10.5% in bronchoalveolar lavage fluid (BALF)

	**LS**	**Non-LS**
**Subjects**	180	68
**Male/female**	111/69	41/27
**Age years**^#^	37 (21–62)	45 (26–72)
**Radiographic stage 0/I/II/III/IV**^#^	0/123/57/0/0	4/16/36/8/4
**Resolving/non-resolving**^#^	166/14	25/43
CD4/CD8 ratio*	9.8 (0.9–56.8)	7.1 (1.2–24.0)
**% Vα2.3 BALF cells**^#^	28.4 (11.0–50.0)	17.8 (11.4–44.3)
HLA-DRB1*03^+^/^−^	155/25	36/32
**Vα2.3 BALF cells %**		
HLA-DRB1*03^+^^#^	29.9	20.5
HLA-DRB1*03^−^**	19.8	14.8
**Patients recovered %**		
HLA-DRB1*03^+^^#^	94	39
HLA-DRB1*03^−^**	80	34

Data are presented as n or mean (range), unless otherwise stated. LS: Löfgreńs syndrome. *: p<0.05, **: p<0.001 and ^#^: p<0.0001, comparing differences between patients with LS and non-LS and for radiological stage differences between stage I and II.

Bronchoscopy with BAL was carried out as described before [8]. Surface markers expressed on T-cells were analysed using flow cytometry and all patients were HLA-typed as previously described [9, 10].

Statistical analyses were performed with Graph Pad Prism 6 (GraphPad Software Inc., San Diego, CA, USA). When comparing several groups such as differences between HLA-DRB1* alleles, p<0.003 (p<0.05 divided by 13) was regarded as significant after Bonferroni correction for the number of alleles (n=13), and otherwise p<0.05 was regarded as significant.

High percentages of CD4^+^ Vα2.3^+^ T-cells (*i.e.* Vα2.3^+^ CD4^+^ T-cells >10.5% in BALF) associated with a resolving disease, as 77% (191 out of 248) of these patients resolved within 2 years compared with 28% (114 out of 413) of patients with normal levels (p<0.0001). The proportion of patients who recovered increased gradually with the increasing proportion of CD4^+^ Vα2.3^+^ T-cells in BALF, for example in patients with 0–5% of CD4^+^ Vα2.3^+^ T-cells 25% had resolving disease; in the range 11–15%, 44% resolved and when there were 21–25% Vα2.3^+^ T-cells, 82% resolved. If >30%, 95% resolved.

Patients with Löfgren's syndrome had higher proportion of CD4^+^ Vα2.3^+^ T-cells in BALF compared to non-Löfgren's syndrome patients and were also younger at disease onset (table 1). Furthermore, patients with Löfgren's syndrome who carried the HLA-DRB1*03 allele had a higher median CD4^+^ Vα2.3^+^ T-cell proportion in BALF compared to HLA-DRB1*03^−^ with Löfgren's syndrome (p<0.0001). Among the HLA-DRB1*03^−^ patients, HLA-DRB1*13 was carried by 88% of the patients with Löfgren's syndrome and by 63% with non-Löfgren's syndrome.

In this study, we chose to focus on patients with an expansion of CD4^+^ Vα2.3^+^ T-cells in BALF. The highest proportion of CD4^+^ Vα2.3^+^ T-cells in the present study was seen in Löfgren's syndrome patients who were HLA-DRB1*03^+^. The non-LS group was characterised by a less pronounced expansion of Vα2.3^+^ T-cells and disease onset at a higher age. That older patients have less favourable outcome has been shown in another cohort [11].

Our hypothesis is that patients with expansion of CD4^+^ Vα2.3^+^ T-cells (*i.e.* Vα2.3^+^ CD4^+^ T-cells >10.5% in BALF) may have a more effective eradication of a presumed disease-promoting antigen. An influx of CD4^+^ Vα2.3^+^ T-cells to the lungs may then explain the concomitant pronounced CD4/CD8 ratio. We have in a previously study showed that the BALF CD4^+^ Vα2.3^+^ T-cells express significantly reduced levels of FOXP3 *versus* CD4^+^ Vα2.3^−^ T-cells [12], suggesting the CD4^+^ Vα2.3^+^ T-cells function as effector cells rather than regulatory cells, in line with a hypothetically more efficient elimination of a hypothetical sarcoidosis-antigen by such T-cells.

The clinical presentation, *i.e.* Löfgren's syndrome or non-Löfgren's syndrome, may reflect an altered immune and inflammatory reaction influenced by different exposures or genetic differences, which also include other inflammatory genes (*e.g.* tumour necrosis factor gene variants linked to HLA-DRB1*03 variants). A hypothetical antigen might itself also have properties that may influence the inflammatory reaction, *e.g.* by inducing auto-immune reactions due to similarities of the inciting antigen and some self-structures or by preferentially stimulating a T helper (Th) 1-, Th 2- or a Th 17-dominant response.

In conclusion, the findings in this study indicate that the more pronounced the expansion of CD4^+^ Vα2.3^+^ T-cells in the BAL fluid is, the better the prognosis. The usefulness of Va2.3^+^ T-cells as a prognostic marker is described here for a Scandinavian cohort. Whether they may be of clinical interest in other populations needs to be analysed in future studies.

## Shareable PDF

10.1183/13993003.01450-2019.Shareable1 This one-page PDF can be shared freely online.
Shareable PDF ERJ-01450-2019.Shareable
