# Supplementary material for: Subpopulations of cells from bronchoalveolar lavage can predict prognosis in sarcoidosis
Source: Eur Respir J. 2020 Jan 30;55(1):1901450. doi: 10.1183/13993003.01450-2019 (PMC6991162; doi:10.1183/13993003.01450-2019)
Supplement: Supplementary file 1 [file ERJ-01450-2019.Shareable.pdf]

AGORA
RESEARCH LETTER

SHAREABLE PDF

Subpopulations of cells from bronchoalveolar lavage can predict prognosis in sarcoidosis

Pernilla Darlington [1], Susanna Kullberg [2], Anders Eklund [2] and Johan Grunewald [2]

Affiliations: [1] Respiratory Medicine Division, Dept of Clinical Science and Education, Södersjukhuset and Karolinska Institutet, Stockholm, Sweden. [2] Respiratory Medicine Division, Dept of Medicine Solna and Center for Molecular Medicine (CMM), Karolinska University Hospital and Karolinska Institutet, Stockholm, Sweden.

Correspondence: Pernilla Darlington, Respiratory Medicine Division, Dept of Medicine, Södersjukhuset, Stockholm, Sweden. E-mail: pernilla.lidin-darlington@sll.se

@ERSpublications
The higher the proportion of a T-cell subset ($CD4^+V\alpha 2.3^+$ T-cells) in bronchoalveolar lavage fluid of sarcoidosis patients, the better the prognosis and therefore it may be used as an additional prognostic tool http://bit.ly/2Mta0Cs

Cite this article as: Darlington P, Kullberg S, Eklund A, *et al*. Subpopulations of cells from bronchoalveolar lavage can predict prognosis in sarcoidosis. *Eur Respir J* 2020; 55: 1901450 [https://doi.org/10.1183/13993003.01450-2019].

This single-page version can be shared freely online.

To the Editor:

Sarcoidosis is characterised by an accumulation of $CD4^+$ T-cells in the lungs and an increased bronchoalveolar lavage fluid (BALF) CD4/CD8 ratio (>3.5) [1]. In sarcoidosis, an expansion of BALF $CD4^+$ T-cells expressing the T-cell receptor Vα2.3 has been associated with good prognosis and with specific HLA-alleles, *i.e.* HLA-DRB1*0301 and HLA-DRB3*0101 (which is often carried together with HLA-DRB1*13). HLA-DRB1*03 and HLA-DRB3*0101 molecules show similarities in the region important for antigen presentation and both may therefore be capable of presenting identical antigens to the lung T-cells [2]. Furthermore, an expansion defined as >10.5% $CD4^+$ $V\alpha 2.3^+$ BALF T-cells is commonly seen in patients with Löfgren's syndrome [3], which is characterised by an acute onset with bilateral ankle arthritis and/or erythema nodosum, bilateral hilar lymphadenopathy and, in some cases, with parenchymal infiltrates and usually fever [4]. We have previously shown that very high expansions of $CD4^+$ $V\alpha 2.3^+$ T-cells are associated with Löfgren's syndrome and a disease duration <2 years [3]. However, not all patients with an expansion of $CD4^+$ $V\alpha 2.3^+$ T-cells have Löfgren's syndrome and resolving disease. In this much enlarged study on a HLA-typed sarcoidosis cohort, we aimed at investigating the clinical characteristics of patients with an expansion of $CD4^+$ $V\alpha 2.3^+$ T-cells in BALF and to analyse if the degree of expansion may predict the prognosis of sarcoidosis.

Copyright ©ERS 2020 This article is open access and distributed under the terms of the Creative Commons Attribution Licence 4.0.
